# Pre-Conditioning Serum Uric Acid as a Risk Factor for Sinusoidal Obstruction Syndrome of the Liver in Children Undergoing Hematopoietic Stem Cell Transplantation

**DOI:** 10.4274/tjh.galenos.2021.2021.0174

**Published:** 2021-12-07

**Authors:** Fatma Visal Okur, Murat Karapapak, Khaled Warasnhe, Umut Ece Arslan, Barış Kuşkonmaz, Duygu Çetinkaya

**Affiliations:** 1 Hacettepe University Faculty of Medicine, Department of Pediatrics, Division of Pediatric Hematology and Pediatric BMT Unit, Ankara, Turkey; 2 Hacettepe University, Institute of Public Health, Department of Health Research, Ankara, Turkey

**Keywords:** Sinusoidal obstruction syndrome, Hematopoietic stem cell transplantation, Uric acid, Inflammation

## Abstract

**Objective:**

Uric acid (UA), a known danger signal released from injured cells, is a valuable sign of inflammation. We aimed to evaluate the association of serum UA levels before the start of conditioning regimens with the risk of hepatic sinusoidal obstruction syndrome (SOS) development after hematopoietic stem cell transplantation (HSCT).

**Materials and Methods:**

Two hundred and twenty-two children who underwent allogeneic HSCT at the Pediatric BMT Unit of Hacettepe University between 2000 and 2014 were included in this retrospective study. Serum UA levels were measured before conditioning as an indicator of the pre-transplant inflammatory status of the patients. Patients with and without a diagnosis of SOS were compared regarding primary diagnosis, previously described risk factors for SOS, and pre-conditioning serum UA.

**Results:**

SOS was diagnosed in 42 patients who had higher pre-conditioning serum UA levels compared to those who did not. Pre-transplant serum creatinine, gamma-glutamyl transferase, bilirubin, ferritin, and C-reactive protein levels did not differ significantly among patients with and without SOS; however, serum albumin was lower in the patients who developed SOS. Receiver operating characteristic analysis revealed that a pre-conditioning UA level higher than 3.32 mg/dL was predictive of SOS. When applied to a multivariate model, only pre-conditioning UA and albumin levels remained significant risk factors for SOS (UA: odds ratio [OR], 2.54; 95% confidence interval [CI], 1.26-5.12, p=0.009; albumin: OR, 0.45, 95% CI, 0.22-0.95, p=0.037).

**Conclusion:**

Our results suggest that pre-conditioning serum UA is an independent risk factor for SOS, and it might be used as an early predictor of hepatic SOS together with previously described clinical and laboratory parameters.

## Abstract (tr)

**Amaç:**

Hasarlı hücrelerden salınan bilinen bir tehlike sinyali olan ürik asit (ÜA), önemli bir enflamasyon belirtecidir. Bu çalışmayı hazırlama rejimine başlamadan önce bakılan serum ürik asit seviyesi ile hematopoetik kök hücre transplantasyonu (HKHT) sonrası hepatik sinüzoidal obstrüksiyon sendromu (SOS) gelişimi riski arasındaki ilişkiyi değerlendirmeyi amaçladık.

**Gereç ve Yöntemler:**

Bu retrospektif çalışmaya Hacettepe Üniversitesi Pediatrik KİT Ünitesi’nde 2000-2014 yılları arasında allojenik HKHT uygulanan 222 çocuk hasta dahil edildi. Serum ÜA seviyeleri, hastaların transplantasyon öncesi enflamatuvar durumunun bir göstergesi olarak hazırlama rejimi öncesi ölçülmüştür. SOS tanısı olan ve olmayan hastalar, birincil tanı, SOS için daha önce tanımlanan risk faktörleri ve rejim öncesi serum ÜA açısından karşılaştırıldı.

**Bulgular:**

SOS tanısı alan 42 hastada rejim öncesi ÜA düzeyleri olmayan hastalara göre daha yüksek saptandı. Nakil öncesi bakılan serum kreatinin, gama-glutamil transferaz, bilirubin, ferritin ve C-reaktif protein parametreler açısından daha düşük olan serum albümini dışında, SOS gelişen hastalar ile gelişmeyen hastalar arasında anlamlı farklılık gözlenmedi. ROC analizi, 3,32 mg/dL’den yüksek rejim öncesi ÜA düzeyinin SOS gelişimini öngördüğünü ortaya koydu. Çok değişkenli bir modele uygulandığında, yalnızca rejim öncesi ÜA ve albümin seviyeleri SOS için önemli risk faktörleri belirlendi (ÜA; odds ratio (OR), 2,54; %95 güven aralığı (GA), 1,26 ila 5,12; p=0,009 ve albumin; OR, 0,45; %95 GA, 0,22 ila 0,95; p=0,037).

**Sonuç:**

Sonuçlarımız, rejim öncesi serum ÜA düzeyinin SOS için bağımsız bir risk faktörü olduğunu ve önceden tanımlanmış klinik/laboratuvar parametreleriyle birlikte hepatik SOS’nin gelişimi açısından erken bir belirteç olarak kullanılabileceğini göstermektedir.

## Introduction

Hepatic sinusoidal obstruction syndrome (SOS), also known as hepatic veno-occlusive disease, is a serious complication of hematopoietic stem cell transplantation (HSCT) and is clinically characterized by fluid retention, painful hepatomegaly, and hyperbilirubinemia. It is the most frequent and well-studied of the early-onset vascular endothelial syndromes that develop after HSCT. It occurs in 5%-15% of patients after allogeneic HSCT. Variations in its incidence are attributed to multiple factors such as the diagnostic criteria used, center experience, year of HSCT, and patient type [1]. Although the reported incidence of SOS has decreased with new transplantation strategies, the severe form of SOS is still associated with significant mortality and early identification of SOS remains challenging [2]. Patient-, disease-, and transplant-related clinical risk factors have been well established, including pre-existing liver dysfunction, disease type and/or disease status, young or old age, allogeneic HSCT, and myeloablative conditioning regimen, but precise prediction of SOS in individuals remains elusive. Early identification and monitoring of high-risk patients using predictive markers will lead to timely treatment implications, which might have a significant impact on survival [3].

SOS is initiated with the sinusoidal endothelial cell damage caused mainly by cytotoxic effects of intensive conditioning regimens including chemotherapy and/or radiotherapy given before transplantation. Prophylactic immunosuppressive therapies, growth factors used to support engraftment, developing infections, and transplantation itself can also cause endothelial damage [4,5]. Clinical evidence suggests that endogenous ‘danger signals’ from injured cells have a role in the pathogenesis of SOS through induction of a non-infectious inflammatory reaction in the allogeneic setting [6]. Uric acid (UA) is an endogenous danger signal released from injured cells that induces the maturation of dendritic cells and expansion of alloreactive T-cells via activation of the NOD-like receptor protein (NLRP)3 inflammasome [7]. Recently, a preclinical study showed the role of NLRP3 inflammasome-mediated IL-1 production in acute graft-versus-host disease (aGVHD) [8]. There is a limited number of clinical studies about the association between serum UA level and aGVHD and their results are controversial [9,10]. The role of serum UA in the pre-transplant period as a risk factor for SOS remains unclear [4,6,11,12]. Although laboratory/clinical markers of endothelial injury preceding SOS development have been described, they have not been adopted to clinical use due to practical limitations and there is still a need for dynamic laboratory parameters that can predict SOS development. Therefore, we evaluated the association between pre-transplant serum UA levels as sensitive markers of inflammation with SOS development after allogeneic HSCT in pediatric patients.

## Materials and Methods

Two hundred and twenty-two children (median age: 7 years, range: 0.3-19; male/female: 150/72) who underwent allogeneic HSCT in the Pediatric BMT Unit of Hacettepe University between 2000 and 2014 were included in the retrospective data analysis. Serum UA levels measured before the initiation of the conditioning regimen (day -9) and after the conditioning regimen was completed (day 0 before transplantation) were analyzed to assess any changes in serum UA that would be indicative of the pre-transplant inflammatory status of individual patients. Pre-conditioning serum creatinine, transaminases, gamma-glutamyl transferase (GGT), and total bilirubin levels were recorded for all patients together with iron overload and cytomegalovirus (CMV) serology for the exclusion of other potential causes of hyperuricemia and hepatic dysfunction. Inflammatory markers (C-reactive protein [CRP], albumin) were also noted at the same time point. The association of serum UA levels of the pre-transplant period with the development of SOS was assessed. HSCT was performed according to standard institutional transplantation procedures. The patients received either myeloablative (intravenous busulfan-based with no area under the curve [AUC] targeting) or reduced-intensity conditioning (fludarabine-based) regimens depending on the primary disease or disease status. GVHD prophylaxis consisted of cyclosporine and methotrexate with or without rabbit anti-thymocyte globulin. Enoxaparin, vitamin E, and ursodeoxycholic acid were administered for SOS prophylaxis to all patients beginning with conditioning. None of the patients received allopurinol before or after transplant. Defibrotide was started when the clinical manifestations of SOS developed with the exclusion of other potential diagnoses. Hepatic SOS was diagnosed when two or more European Society for Blood and Marrow Transplantation (EBMT) diagnostic criteria (refractory thrombocytopenia, weight gain >5% above baseline, hepatomegaly, ascites, and bilirubin value ≥2 mg/dL) were present and the EBMT severity grading system was used [12,13]. The study was approved by the institutional ethics committee.

The associations between clinical variables including patient- and transplant-related risk factors (primary disease, conditioning regimen, HLA compatibility, CMV status, ferritin, GGT, pre/post-conditioning serum UA levels, albumin, and CRP) and hepatic SOS were analyzed using a logistic regression model. Only the variables with p<0.2 in the univariate analysis were subjected to multivariate analysis. The variables with p<0.05 were considered significant. Comparisons within and between the groups were performed using the Wilcoxon signed-rank and Mann-Whitney U test, respectively. Median follow-up time and overall survival were estimated using Kaplan-Meier limit estimation. The Cox proportional hazards model for multivariate analyses of survival was used. Receiver operating characteristic (ROC) curve analysis was performed to calculate sensitivity, specificity, and AUC for peak pre-conditioning serum UA of ≥3.32 mg/dL.

## Results

The patient and transplant characteristics are summarized in Table 1. There were 222 children enrolled in the study with a median age of 7 years (range: 0.3-19). Sixty-eight percent of the children were male and most patients were transplanted for non-malignant diseases (69%), mainly non-malignant hematological diseases and primary immunodeficiencies, while the rest of the patients were transplanted for hematological malignancies (31%). Most patients received a bone marrow graft (71%) from an HLA-matched family donor (89%) following myeloablative conditioning (70%). Median values of pre-transplant serum creatinine, transaminases, and total bilirubin levels were all within the normal range for age (Table 1).

When the median serum UA levels of all patients were evaluated, pre-conditioning UA levels were found to be significantly higher than the post-conditioning levels (3.1 mg/dL, range: 0.68-6.9 at day -9 vs. 2.8 mg/dL, range: 0.61-6.94 at day 0 post-conditioning; p=0.02). After grouping patients by the development of hepatic SOS, the 42 patients (19%) who developed mostly mild/moderate hepatic SOS (91%) were found to have higher median pre-conditioning UA levels compared to those who did not develop SOS (3.6 mg/dL vs. 3.0 mg/dL; p=0.01). Pre-transplant serum creatinine, GGT, total bilirubin, ferritin, CRP, and albumin were checked, and while most of these variables did not differ significantly regarding SOS groups (p>0.05), albumin was found to be lower in patients with SOS compared to those without SOS (p=0.02) (Table 2). Among the 42 patients diagnosed with SOS, 25 (60%) had mild, 13 (31%) had moderate, and 4 (9%) had severe disease. Thirty patients out of 42 recovered from SOS, while the 4 patients with severe SOS and 8 patients with other early transplant-related complications died.

While a significant decrease in the serum UA levels of the patients who did not develop SOS was observed on the day of HSCT following conditioning (3.05±0.09 mg/dL vs. 2.89±0.09 mg/dL; p=003), there was no significant change in the serum UA levels of patients who developed SOS (3.75±0.25 mg/dL vs. 3.2±0.18 mg/dL; p>0.05). The development of SOS was associated with higher pre-conditioning UA levels (p=0.002). The ROC curve was drawn to determine a cutoff value and evaluate the predictive value of pre-conditioning UA for hepatic SOS development. As shown in Table 3, the cutoff value of UA in the pre-transplant period before the start of conditioning for hepatic SOS was 3.32 mg/dL with an AUC of 62.4%, sensitivity of 62%, and specificity of 61%. Hence, the pre-conditioning UA level seems to be predictive of hepatic SOS. We observed a difference in the frequency of SOS among the patients when compared regarding the UA cutoff value (above the cutoff: 26.8% vs. below the cutoff: 12.8%; p=0.008) Next, we conducted univariate analysis to investigate the associations among pre-conditioning serum UA, previously described risk factors, and hepatic SOS, as illustrated in Table 4. When applied to a multivariate model, pre-conditioning UA remained significant as a risk factor for SOS (UA: OR, 2.54; 95% CI, 1.26-5.12; p=0.009). The odds of SOS incidence in patients with UA higher than 3.32 mg/dL were 2.5 times higher than among patients with UA below the cutoff. Pre-transplant serum albumin was also associated with SOS (OR, 0.45; 95% CI, 0.22-0.95; p=0.037) among all other risk factors included in the model.

The probability of 10-year overall survival after HSCT was 75% for all patients enrolled with a median survival of 114 months. There was a significant difference between survival rates of patients who developed SOS (64%; mean survival: 77.5±8.5 months) and those who did not (77%; mean survival: 82±3.2 months) (p=0.047). However, the cutoff value of pre-conditioning UA of 3.32 mg/dL had no significant effect on the survival of the patients (OR, 0.86, 95% CI, 0.63-1.18; p*=*0.35).

## Discussion

SOS of the liver is thought to result from conditioning regimen-related cytotoxic injury to the hepatic sinusoidal endothelium and hepatocytes intensified by cytokine-mediated alloimmunity. Although different combinations of biomarkers for endothelial injury and hemostasis have been described to predict the occurrence of SOS by various studies, early and precise prediction of SOS is still challenging, probably due to the lack of well-defined specific marker panels [14,15]. In the present study, we aimed to retrospectively investigate the association of serum UA levels measured before initiation of the conditioning regimen with hepatic SOS in 222 pediatric patients who underwent allogeneic HSCT. Our results indicate that high pre-conditioning serum UA level is an independent pre-transplant risk factor for SOS development after allogeneic HSCT.

**Figure 1:**
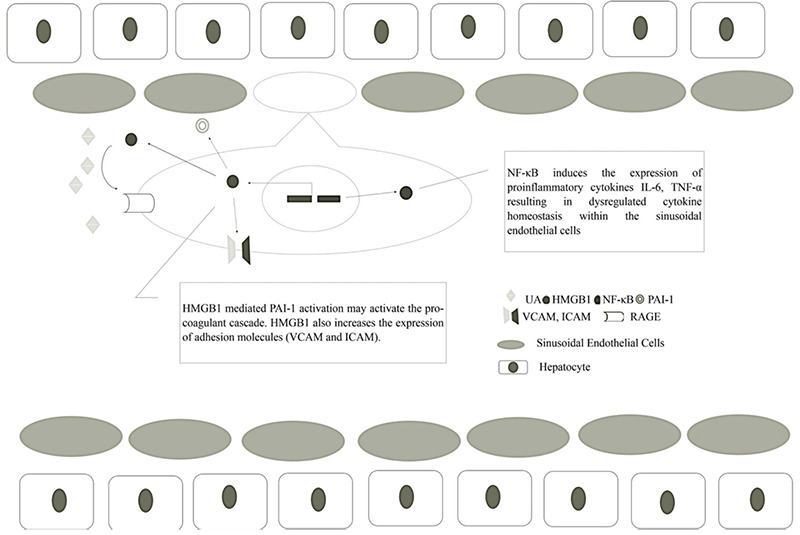
Potential explanation for the role of uric acid in the development of SOS in the inflammatory setting. High levels of uric acid activate the RAGE/HMGB1 signaling pathway in sinusoidal endothelial cells and increase NF-κB expression. NF-κB overexpression increases the secretion of pro-inflammatory cytokines, i.e. TNF-α and IL-6, from damaged sinusoidal endothelial cells, resulting in cytokine derangement within the hepatic sinusoids leading to immune activation. HMGB1 also increases the expression of adhesion molecules (VCAM and ICAM) and PAI-1, resulting in the activation of the pro-coagulant cascade and sinusoidal obstruction [24,27]. SOS: Sinusoidal obstruction syndrome; RAGE: receptor for advanced glycation end products; HMGB1: high mobility group box chromosomal protein 1; IL-6: interleukin-6; TNF-α: tumor necrosis alpha; NF-κB: nuclear factor κB; ICAM: intercellular adhesion molecule; VCAM: vascular cell adhesion protein; PAI-1: plasminogen activator inhibitor-1.

Even though danger-associated molecular patterns released from injured cells including extracellular adenosine triphosphate, high mobility group box chromosomal protein 1, and UA are recognized to play a role in aGVHD pathogenesis, the role of UA, as a pro-inflammatory mediator in allogeneic immune responses, is still ambiguous [11] (Figure 1). A phase I study reported that patients with aGVHD had higher serum UA levels during the pre-transplant period compared to patients without aGVHD [10]. The incidence of grade II to IV aGVHD was significantly decreased in the group treated with urate oxidase during conditioning. On the contrary, a retrospective study indicated a significant association between aGVHD and low serum UA levels [9]. The discrepancies among previous reports on the role of UA in the development of aGVHD probably arise from both the paradoxical actions of UA as a pro- and anti-oxidant [16] and its complex role in inflammation. Preclinical studies suggest that decreasing serum UA levels during conditioning before HSCT may suppress recipient antigen-presenting cell activation and T-cell response [7,17]. Thus, considering the current understanding of SOS pathogenesis, UA, as an endogenous danger signal released from injured cells, seems to be an attractive target for SOS prediction and preventive measures. There is no previous preclinical or clinical study that investigated the impact of UA as a pro-inflammatory mediator on SOS and its predictive role. Thus, it would be reasonable to consider that a common mechanism of initiation and/or maintenance of inflammation through endogenous ‘danger signals’ from injured cells underlies the development of SOS after HSCT.

The results of this study support our initial hypothesis about the association of high pre-transplant serum UA levels with SOS and are in parallel with previous reports about the significant role of high UA levels in transplant outcomes such as aGVHD and survival [18,19,20]. Unlike most other studies assessing the prognostic value of several biomarkers at different time points after transplantation, either before or during the early stage of hepatic SOS, we preferred to evaluate serum UA levels before initiation of conditioning, which could be a critical period for the early detection of the inflammatory background of individual patients. Interestingly, serum UA levels were higher in the patients with SOS even before the start of the conditioning regimen compared to patients without SOS. In addition, the UA levels decreased after conditioning in the patients without SOS, while they remained high in those with SOS. This seems to conflict with previous studies reporting elevated serum UA levels following conditioning because of its cytotoxicity [10,21]. High pre-conditioning UA levels that remained unchanged following conditioning together with low serum albumin levels in our patients with SOS might be attributed to ongoing subtle inflammation, occult tissue injury, and accelerated cell turnover related to primary disease/disease status, previous therapies, and infections [22]. Post-conditioning decrease in UA levels in patients without SOS might be explained by the suppressive effect of myeloablative/lymphodepleting conditioning regimens on the recipient’s immune system, indicating the presence of a fine balance between pro-inflammatory and anti-inflammatory mechanisms during the peri-transplant period. Newly developing bone marrow aplasia could also contribute to this change in UA levels. Haen et al. [23] reported that serum UA levels remained low until incipient hematopoietic recovery in HSCT patients and leukemia patients undergoing induction chemotherapy. They emphasized the role of UA as a potential marker for bone marrow activity during aplasia, besides its role in immune activation and inflammation [23].

Endothelial damage related to conditioning, acting as a second hit, potentiates immune activation leading to alloimmunization and the development of SOS. Dysregulation of cytokine homeostasis is common after conditioning. Pro-inflammatory cytokines including tumor necrosis alpha, interleukin (IL)-1, and IL-6 have been reported to activate xanthine oxidase, thus stimulating UA production, which is involved in the pathogenesis of early non-infectious transplant complications, such as SOS. Even in the absence of clinically defined hyperuricemia (serum UA of >7 mg/dL) based on the solubility limit of urate in body fluids, this positive feedback loop may cause a further release of cytokines and endogenous adjuvants that contribute to the development of endothelial cell injury in an inflammatory setting [24,25], as in the case of our patients with high pre-conditioning UA who developed hepatic SOS over the transplant course. There are several reports investigating the association of serum UA and transplant outcomes, which have defined their own cutoff values for their own patient cohorts [18,26]. Our determined cutoff value of 3.32 mg/dL for pre-conditioning UA was derived from ROC analysis and it was predictive of hepatic SOS development after allogeneic HSCT. Multivariate analysis confirmed the association of serum UA higher than 3.32 mg/dL with the risk of hepatic SOS. Low serum albumin in the pre-transplant period was also identified as a risk factor for SOS. Serum albumin serves as a laboratory marker of inflammatory status, and the prognostic value of low serum albumin for transplant outcomes has been revealed by previous studies.

### Study Limitations

One of the limitations of our study is its retrospective nature. Therefore, serial measurements of serum UA and other pro-inflammatory and/or endothelial injury markers over the transplant course could not be performed. Also, the design of our study did not allow us to investigate whether UA plays an active role in the pathogenesis of hepatic SOS through induction of a non-infectious inflammatory reaction in the allogeneic setting or is simply a biomarker of inflammatory status. Since our patient population mostly included pediatric patients who received bone marrow grafts from matched related donors after myeloablative conditioning for non-malignant disorders, we could not draw any conclusion about the impact of serum UA on SOS development in different transplant settings such as unrelated and haploidentical transplants, which carry a higher risk for SOS. However, we think that the association of serum UA higher than the cutoff value with SOS in such a restricted population could be accepted as proof of its predictive strength.

## Conclusion

This is the first report about the association of pre-conditioning serum UA level and hepatic SOS in HSCT recipients and it supports the use of pre-transplant serum UA level as a risk factor for SOS. There is a need for mechanistical studies to understand the precise role of UA in the pathogenesis of SOS as an inflammatory mediator in the allogeneic transplant setting. Further studies with independent cohorts and in different transplant settings may also help to clarify the role of UA in predicting high-risk patients for SOS together with other defined clinical and laboratory markers of endothelial injury.

## Figures and Tables

**Table 1:**
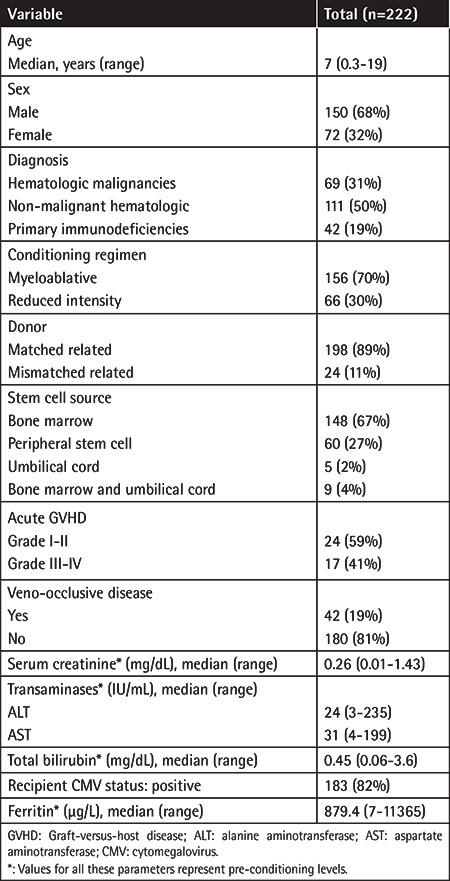
Patient and transplant characteristics.

**Table 2:**
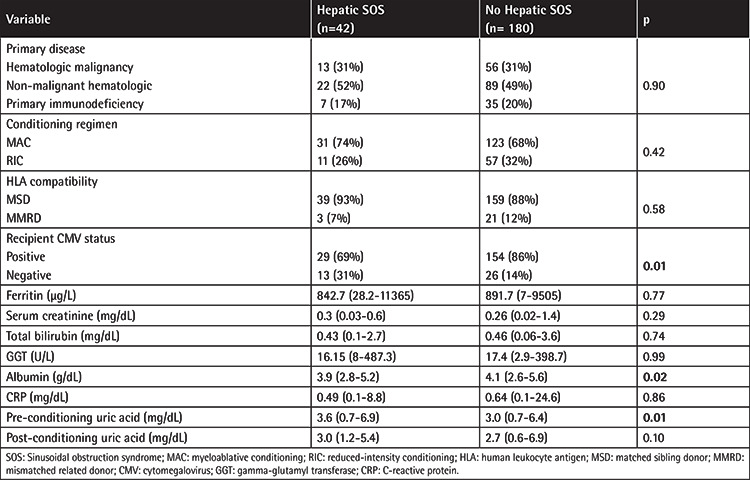
Comparison of patients with and without hepatic sinusoidal obstruction syndrome.

**Table 3:**
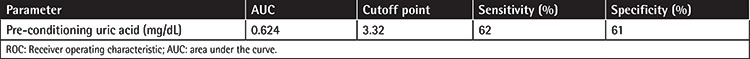
ROC analysis for pre-conditioning uric acid.

**Table 4:**
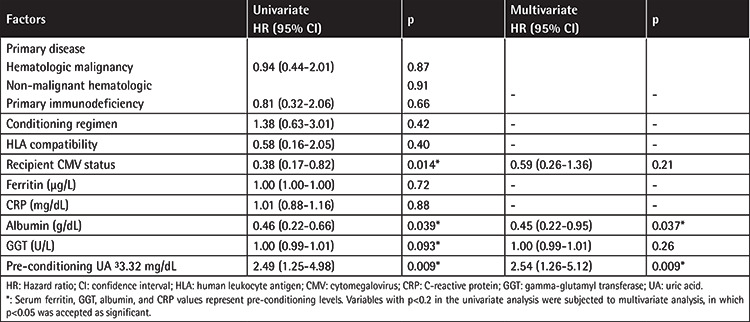
Risk factors for hepatic sinusoidal obstruction syndrome by logistic regression analysis.
